# Structural Bases of Norovirus RNA Dependent RNA Polymerase Inhibition by Novel Suramin-Related Compounds

**DOI:** 10.1371/journal.pone.0091765

**Published:** 2014-03-12

**Authors:** Romina Croci, Margherita Pezzullo, Delia Tarantino, Mario Milani, Shwu-Chen Tsay, Radhakrishnan Sureshbabu, Yi-Jin Tsai, Eloise Mastrangelo, Jacques Rohayem, Martino Bolognesi, Jih Ru Hwu

**Affiliations:** 1 Department of BioSciences, University of Milano, Milano, Italy; 2 Istituto di Biofisica,CNR IBF, Milano, Italy; 3 Department of Chemistry, National Central University, Jhongli, Taiwan; 4 Department of Chemistry and Frontier Research Center on Fundamental and Applied Sciences of Matters, National Tsing Hua University, Hsinchu, Taiwan; 5 Riboxx GmbH, Pharmapark Radebeul, Radebeul, Germany; 6 Institute of Virology, Dresden University of Technology, Dresden, Germany; University of Washington, United States of America

## Abstract

Noroviruses (NV) are +ssRNA viruses responsible for severe gastroenteritis; no effective vaccines/antivirals are currently available. We previously identified Suramin (**9**) as a potent inhibitor of NV-RNA dependent RNA polymerase (NV-RdRp). Despite significant *in vitro* activities *versus* several pharmacological targets, Suramin clinical use is hampered by pharmacokinetics/toxicity problems. To improve Suramin access to NV-RdRp *in vivo*, a Suramin-derivative, **8**, devoid of two sulphonate groups, was synthesized, achieving significant anti-human-NV-RdRp activity (IC_50_ = 28 nM); the compound inhibits also murine NV (mNV) RdRp. The synthesis process led to the isolation/characterization of lower molecular weight intermediates (**3**–**7**) hosting only one sulphonate head. The crystal structures of both hNV/mNV-RdRps in complex with **6**, were analyzed, providing new knowledge on the interactions that a small fragment can establish with NV-RdRps, and establishing a platform for structure-guided optimization of potency, selectivity and drugability.

## Introduction

**Figure 1:**
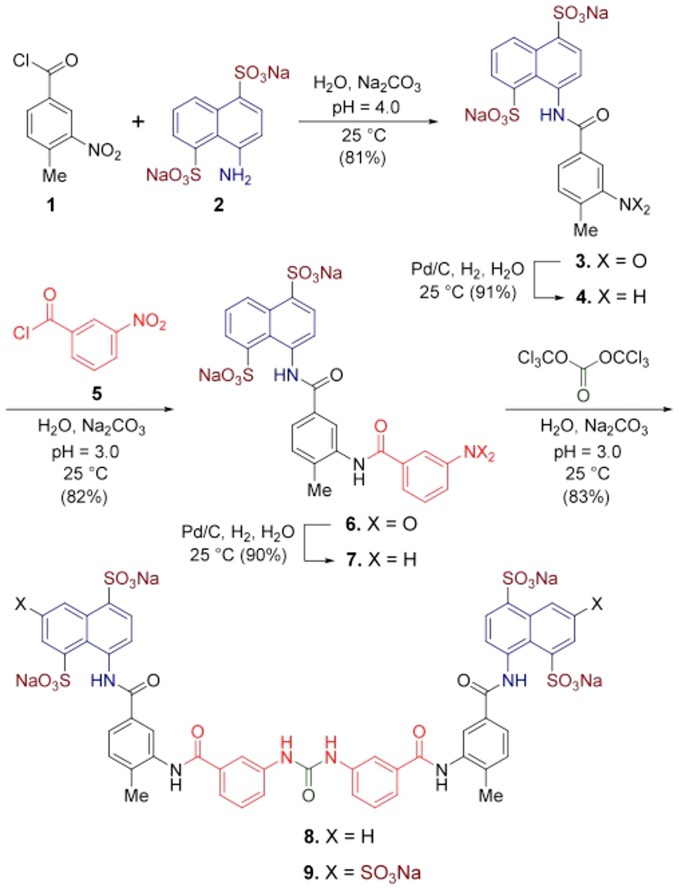
Suramin derivative 8 synthesis. Total synthesis of Suramin derivative **8** from commercially available starting materials. The Suramin molecule (**9**) is also shown for comparison.

Norovirus (formerly Norwalk-like virus, NV) is a genus of the *Caliciviridae* family that is a major causative agent of non-bacterial acute gastroenteritis in humans. Outbreaks commonly occur in settings such as hospitals, nursing homes, cruise ships, university dormitories, and military barracks. It is estimated that NV infection may account for up to 200,000 deaths per year in infants and young children of the developing countries [1], [2]. Currently, no vaccines [3], [4] or specific antiviral agents are available to combat NV; thus, there is an urgent and still unmet need for discovery and development of broad spectrum small-molecule therapeutics against this severe pathogen. Human NVs are fairly species-specific and do not appear to infect small animals, even if animal models are under development [5], [6], [7]. A widely used model system shedding light on NV pathogenesis and replication strategies is the murine model of NV infection, obtained by infecting mice with murine NVs (mNVs) [8]. The calicivirus genomes consist of a single stranded, positive-sense poly-adenylated RNA molecule that averages 7500 nucleotides in length. It is organized in either two or three open reading frames (ORF-1 to ORF-3), depending on the particular genus. ORF-1 is predicted to encode a single polyprotein that, after co-translational processing by the viral protease, results in the nonstructural proteins required for replication of the viral genome [9], [10] and their precursors [11]. Among these, nonstructural protein 7 [RNA-dependent RNA polymerase (RdRp) domain] plays a key role in genome replication, as well as in the synthesis and amplification of additional subgenomic RNA [12]. Notably, since RdRp is not present in mammalian cells, it appears as a suitable target for inhibition in the context of antiviral prophylaxis. Suramin (**9**, Fig. 1) is a polysulphonated naphthylurea, which has been used as the drug of choice for treatment of African trypanosomiasis and onchocerciasis since 1924 [13]. Different Suramin applications have been reported, including inhibition of reverse transcriptase [14], P2X and P2Y nucleotide receptor family antagonism [15], [16], and blocking actions on various growth factors [17]. Also, since Suramin hinders cell proliferation and migration, as well as the formation of new blood vessels, it has been tested for potential use as an anticancer agent [18]; moreover, Suramin was noted to induce hyperglycaemia [19], [20]. Several clinical trials based on Suramin and Suramin-like compounds have nevertheless proven unsatisfactory, as *in vitro* results did not translate into the desired clinical response *in vivo*. Except for trypanosomiasis, in fact, all other trials did not reach the clinical level due to challenges related to Suramin pharmacokinetics and toxicity [20], [21], [22]. We previously identified Suramin as a potent inhibitor of hNV and mNV-RdRps, and solved the crystal structures of mNV RdRp in the absence/presence of Suramin, highlighting an inhibitor binding site located between the RdRp fingers and thumb domains. In particular, the cleft occupied by the inhibitor is lined with conserved amino acids, likely building the access route for the incoming NTP that will be linked to the nascent RNA chain. [23]. Due to the high potency of Suramin in enzymatic assays, its optimization into a more drug-like compound would not require enhancing its interaction with the therapeutic target (*e.g*. hNV-RdRp), rather it should improve the ability of Suramin to reach the therapeutic target *in vivo*. Suramin is a polar compound, with log *P* value lower than 0; thus, it is likely to traverse the epithelium slowly *via* paracellular channels. In addition, it should be recalled that Suramin's high molecular weight (1429 Da) might promote biliary excretion, reducing its overall systemic bioavailability [24], [25].

The crystal structure of mNV-RdRp in complex with Suramin showed that only two of the three sulphonate groups on the Suramin naphthalene rings establish ionic interactions with basic residues of the enzyme [23]. On the basis of such structural information we undertook the chemical synthesis and biochemical characterization of carbamide **8** (see Fig. 1), a Suramin derivative bearing only two sulphonate groups on each naphthalene ring, as a first optimization step. We then characterized **8**, together with lower molecular weight synthetic reaction intermediates, in enzymatic inhibition assays versus hNV and mNV RdRps. To further address the inhibitory mechanistic issues, and to gather new information for rational drug design, we then analyzed the crystal structures of both hNV and mNV-RdRps in their complexes with diamide **6**, one of the reaction intermediates hosting only one sulphonate head, showing a favorable log *P* value relative to Suramin.

Aiming to further develop anti-norovirus compounds, we report here the details of the synthetic steps to produce five inhibitors, the analysis of their hNV and mNV-RdRp inhibitory activities, together with the crystal structure analysis of hNV and mNV-RdRps in their complexes with **6**, a low molecular weight representative compound in this class.

## Results

### Syntheses and Spectral Characteristics of Carbamide 8

For examination of the effects resulting from the sulphonate group (in position 3) of compounds in the family of Suramin (**9**), a total synthesis of carbamide **8** was performed as shown in Fig. 1. The condensation of commercially available nitrobenzoyl chloride **1** with sodium naphthalene disulphonate **2** in water at pH 4.0 yielded nitroamide **3** in 81% yield. Reduction of the nitro group in compound **3** with hydrogen gas in the presence of Pd catalyst led to the corresponding aniline **4** in excellent yield; meanwhile, the two aryl groups remained intact. A repeat of the same type of condensation reaction by use of aniline **4** and benzoyl chloride **5** afforded the desired diamide **6**, which was further hydrogenated to give stable sodium salt of aniline **7**. Solubility of this compound was found very poor in most of organic solvents, including THF and DMSO. The carbonylation of aniline **7** with 1, 1′-carbonyldiimidazole (CDI) [26] in a mixture of water and toluene in different ratios at room temperature or under reflux conditions did not lead to the desired carbamide **8**. Instead, the starting materials remained intact. This problem was circumvented by use of triphosgene in a slight excess to react with aniline **7** at pH 3.0 in an aqueous Na_2_CO_3_ solution containing toluene. Accordingly, **8** was generated as a white powder in 83% yield.

The structures of sulphonates **3**–**8** were confirmed by their spectroscopic characteristics. For instance, the exact mass of carbamide **8** (C_51_H_36_N_6_Na_4_O_17_S_4_+2 Na)^2+^ was measured by the electrospray ionization (ESI) method as 635.0116, which is very close to its theoretical value of 635.0197. Its ^13^C NMR spectrum displayed 26 peaks as expected; among which the peak with *δ* = 153.53 was attributed to the carbamido carbon (*i.e.* NCON). Two peaks at *δ* = 165.81 and 165.06 in the downfield region belonged to the two different amide carbons. Its ^1^H NMR spectrum showed nine sets of peaks, which resonated between 9.09–7.37 ppm with singlet, doublet, and triplet. They were associated with the aromatic protons with the expected splitting pattern for carbamide **8**. Its IR spectrum exhibited strong absorptions at 1652 and 1634 cm^−1^, which were attributed to the stretching vibration of an amido carbonyl group.

### Physical and Biological Properties of Sodium Organosulfonates 3–8 and Suramin (9)

Bookser's method [27] was applied to obtain the water solubility of sodium organosulphonates **3–8** and Suramin (**9**) by use of HPLC with a UV detector (see Table 1). Furthermore, the “shake–flask method” [28] was applied to obtain their hydrophobicity by use of *n*-octanol and water. The apparent partition coefficient (*P*) shown in Table 1 is the ratio of the concentration in *n*-octanol to the total concentration (*i.e.*, ionized plus non-ionized) of the species in the aqueous phase, [29].

**Table 1:** Water solubility and hydrophobicity of organic sulphonates **3–8** and Suramin.

Compound	water solubility^a^ (mg/ml)	hydrophobicity^b^ log *P*
**3**	260	−2.19
**4**	339	−2.33
**6**	179	−1.64
**7**	249	−2.11
**8**	114	−1.61
Suramin (**9**)	138	−3.42

a The Bookser's method was applied.

b The shake–flask method was applied.

### Enzymatic assays

hNV and mNV-RdRp inhibition assays were performed monitoring the impairment of RNA synthesis as a function of inhibitor concentration, as previously described [23]. Briefly, RdRp activity was assessed *in vitro* (measuring PicoGreen fluorescence) following the synthesis of double-stranded RNA from a single-stranded RNA poly(C) template annealed with a G_12_ primer. The assay can easily be adapted for any viral RNA polymerase [30]. Under our experimental conditions, **8** inhibited hNV-RdRp with the same IC_50_ value of Suramin (∼30 nM), whereas **6** and other synthetic intermediates proved to be ∼30–35-fold less potent (Table 2). All the low molecular weight compounds (**3**–**7)** are generally more potent inhibitors of mNV rather than of hNV-RdRp (Table 2).

**Table 2:** IC_50_ values of Suramin derivatives and Suramin (**9**) against mNV and hNV-RdRps.

	IC_50_ (nM)	
Compound	mNVRdRp	hNVRdRp
**3**	200±10	1280±70
**4**	160±7	1100±200
**6**	115±15	1000±90
**7**	160±6	1100±50
**8**	60±4	28±4
Suramin (**9**)	70±3	27±3

### Thermofluorimetric assays

In order to exclude that **8** and **6** inhibition might be linked to some form of RdRp destabilization/aggregation/denaturation, a process generally reflected by a variation of the protein melting temperature (T_m_), we performed thermofluorimetric assays on free and inhibited NV-RdRps. All the acquired data showed that NV-RdRps displays similar melting temperatures in the absence (hNV/mNV-RdRp Tm = 41.5/39.6 ±0.2 °C), or in the presence of **6** (hNV/mNV-RdRp Tm = 41.2±0.2/39.4±0.3 °C), or of **8** (hNV/mNV-RdRp Tm = 41.4/39.8 ±0.2 °C), thus proving that enzyme inhibition is not caused by denaturation of the protein.

### Crystal structures of NV-RdRps bound to 6

To shed light on the mechanisms of RdRp inhibition exerted by the organic sulphonate compounds described above, we undertook crystallographic analyses of both enzymes in their complexes with **6**. To address the crystal structures of the **6** adduct, hNV-RdRp and mNV-RdRp crystals were soaked in stabilizing solutions of **6** at concentration of 10 or 20 mM (see Materials and Methods for details).

**Figure 2:**
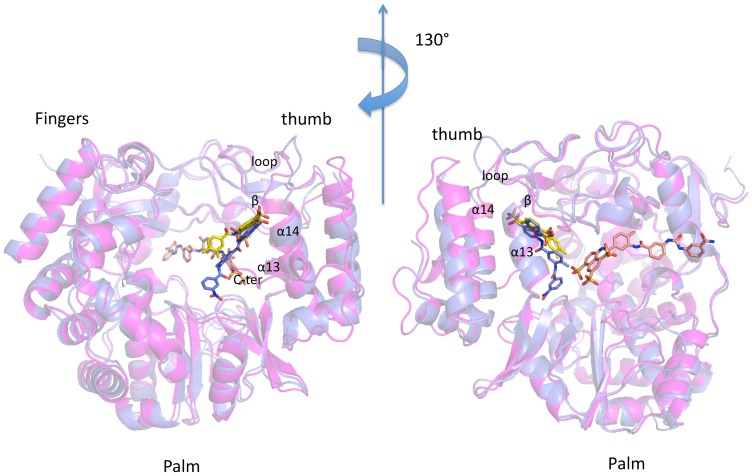
Superposition of hNV and mNV-RdRp/6 complex structures. Superposition of the crystal structures of hNV-RdRp in cartoon (magenta) bound to **6** (yellow carbon atoms) in sticks, onto mNV-RdRp in cartoon (blue), bound to **6** (carbon atoms in green) in sticks. The suramin position (in sticks orange carbons) is obtained from superposition of pdb-id 3UR0 (Mastrangelo et al., 2012) (Figures created using PyMol (http://www.pymol.org)).

**Figure 3:**
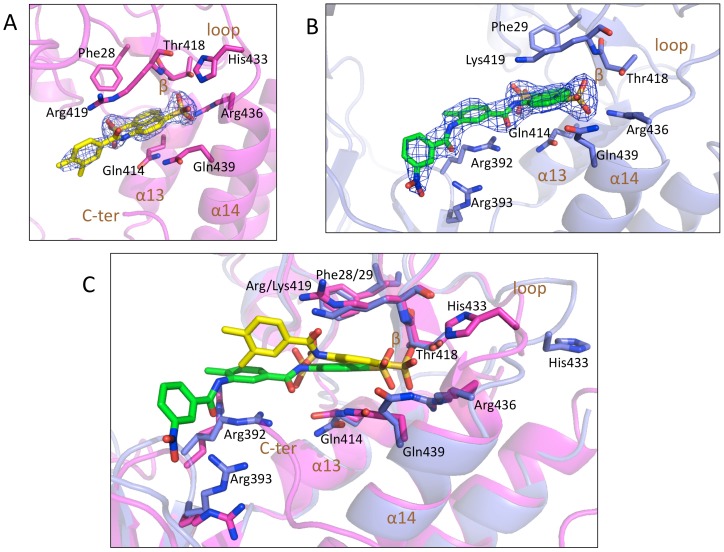
Interaction network of hNV and mNV-RdRp/6 complex. A) Fragment of **6** (carbon atoms in yellow) bound to hNV-RdRp in cartoon (magenta). All the amino acids involved in interaction with the inhibitor molecule are shown in sticks (carbon atoms in magenta). B) **6** (carbon atoms in green) bound to mNV-RdRp in cartoon (blue). All the amino acids (carbon atoms in blue) involved in interaction with the molecule are shown in sticks. 2Fo-Fc electron density contoured at 1 sigma in blue grid. C) Superposition of hNV-RdRp and mNV-RdRp, showing the structures of **6** (carbon atoms in yellow/green, respectively) bound to hNV-RdRp and to mNV-RdRp (cartoon in magenta/blue, respectively). The interacting amino acids are shown in sticks (magenta/blue carbon atoms, respectively).

*hNV-RdRp/* ***6*** diffraction data were collected at 100 K at the ESRF beam line ID29 (Grenoble, France); the crystal structure was solved by molecular replacement (MR) and refined to final crystallographic R-factor/R-free values of 16.4/20.9%, at 2.02 Å resolution (Table 3). After the first refinement cycles residual electron density was visible in a positively charged cleft within the polymerase thumb domain, in a location different from the Suramin binding site [23] (Fig. 2). The naphthalene sulphonate head of **6,** the linked amido group and the phenyl methyl group were accordingly modeled in the new site formed by the α13-β-loop-α14 secondary structure elements (Fig. 2). However, density for the remaining part of the compound was not visible, as confirmed by an independent dataset collected after soaking hNV-RdRp crystals at 20 mM concentration of **6**. The naphthalene ring of **6** is sandwiched between the amido groups of Gln414/Gln439 on one side, and Phe28/Arg419, on the other. The negative charges of the two sulphonates are balanced by residues Arg419, Arg436, and likely (considering that the soaking took place at pH 6) by His433. In particular the sulphonate in position 1 is highly stabilized by an electrostatic interaction network involving the side chains of Thr418, His433, Arg436, and the main chain nitrogen of Arg419 (Fig. 3A). The unmodeled tail of **6** would be hosted in the polar central region of the enzyme which accommodates dsRNA during elongation, wide enough to allow conformational flexibility of the second half of the inhibitor, in keeping with the lack of an interpretable electron density signal.

**Table 3:** X-ray data-collection and refinement statistics for the hNV-RdRP/**6** complex.

Protein crystals	*hNV-RdRp/6*	*mNV-RdRp/6*
**6** concentration (mM)	10	20
Resolution (Å)	71.80–2.02	64.96–2.30
Space group	I222	P2_1_
Unit-cell parameters (Å, °)	*a* = 89.2; *b* = 112.0; *c* = 121.1	*a* = 109.2; *b* = 162.4; c = 123.0 *β* = 97.0°
Molecules in a.u.	1	6
Mosaicity (°)	0.2	0.3
Unique reflections	39,356 (5,729)*	188,102 (27,425)*
Completeness (%)	98.8 (99.1)	99.9 (99.8)
Redundancy	3.1 (3.2)	3.2 (3.2)
Rmerge† (%)	8.4 (30.5)	9.8 (72.0)
Average *I*/σ (*I*)	8.4 (3.4)	7.7 (1.6)
R factor‡/Rfree§ (%)	16.4/20.9	19.1/25.4
r.m.s.d. bonds (Å)	0.011	0.010
r.m.s.d. angles (°)	1.48	1.33
Average protein *B* factors (Å^2^)	28.2	A = 38.4, B = 39.9, C = 42.0, D = 42.9, E = 49.5, F = 58.4
Average ligand *B* factors (Å^2^)	55.6	A = 78.2, B = 58.9, C = 70.2, D = 67.3, E = 85.5, F = 87.7
Residues in most favored regions (%)	95.0	92.9
Residues in additionally allowed regions (%)	5.0	7.1
PDB	4NRT	4NRU

*Values in parentheses are for the highest resolution shell: (2.13–2.02 Å for hNV RdRp/**6**, 2.42–2.30 Å for mNV RdRp/**6**).

† R_merge_ = Σ |*I*−(*I*)|/Σ *I* x 100, where *I* is intensity of a reflection and (*I*) is its average intensity.

‡ R _factor_ = Σ |F_o_−F_c_|/Σ |F_o_| x 100.

§ R_free_ is calculated on 5% randomly selected reflections, for cross-validation.

*mNV-RdRp/* ***6*** diffraction data were collected at 100 K at the ESRF beam line ID29 (Grenoble, France); the crystal structure was solved by MR (2.3 Å resolution) and refined to final crystallographic R-factor/R-free values of 18.5/24.8% (see Materials and Methods and Table 3). Six mNV-RdRp molecules are hosted in the crystal asymmetric unit. Inspection of the refined model shows that the naphthalene disulphonate head of the inhibitor binds to the same RdRp location identified for hNV-RdRp (Fig. 2). In the murine protein compound **6** could be fully modeled in all the subunits of the crystal asymmetric unit (Fig. 3B).

Relative to the hNV/**6** binding mode described above, in mNV RdRp the naphthalene ring of the molecule is found slightly repositioned, showing a rotation of the naphthalene plane of about 20° toward the palm domain, and a translation away from the α14 helix (by 1.3 Å, Fig. 3C). The observed roto-translation of the ligand is related to the loss of the interaction with Gln439 and with His433, both pointing toward the solvent in mNV RdRp (Fig. 3C). Such shift of the ligand molecule promotes the stacking interaction of the toluene group of **6** with Arg392 guanidine moiety (H-bonded to **6** carbonyl, and in electrostatic interaction with 2 sulphonates), which allows the stabilization of the entire inhibitor (Fig. 3B, C).

## Discussion

For the successful synthesis of sodium sulfates **3**, **6** and **8**, it was crucial to maintain the pH values of the media throughout the entire processes at 4.0 for conversion of **2→3**, and at 3.0 for the **4**→**6** and **7**→**8** conversions. An aqueous Na_2_CO_3_ solution (2.0 M) was frequently added to the reaction mixtures; otherwise, the pH values would be reduced to ∼1 during the course of the reactions. High care was taken during the coupling of aniline **7** with triphosgene, which may decompose to an insidious poison phosgene gas during the carbonylation reaction. Accordingly, control of its pH value and addition of a Na_2_CO_3_ solution were performed in a closed system by a syringe. Although phosgene can be used in the synthesis of urea by reacting with amines [24], two advantages are associated with triphosgene: *i*) being a solid, triphosgene is safer to handle than phosgene, which is a gas or a toluene solution; *ii*) a stoichiometric amount of triphosgene suffices for urea formation, which proceeded in a good yield. Solubility of chemical entities in water plays an important role in their development as a lead for new drugs [24] and is related to the structure–activity relationship. Poor water solubility of a compound may result in decreased oral absorption regardless of its good permeation rate across the intestinal mucosa into the circulation. The apparent partition coefficient (*P*), which is a useful parameter for understanding the behavior of drug molecules, was measured. Often it is used to predict the distribution of a drug compound in a biological system, the log *P* value being related to absorption, excretion, and penetration [31]. The measurement of the apparent partition coefficient provides a simple “*in vitro*” method to predict the behavior of a compound in the body and to select the most promising drug candidates from a large pool of ionizable compounds. Thus the log *P* of thousands of drugs and potential drugs has been measured over the years. Our results presented in Table 1 indicate that the water solubility fell into the range of 114–339 mg/ml for compounds **3**–**8**. Carbamide **8** containing four hydrophilic sulphonate groups was less water soluble than Suramin (114 versus 138 mg/ml), which contained six sulphonate groups. The new **8** also showed a better hydrophobicity than Suramin (log *P* –1.61 versus –3.42).

It is important to notice that in the DrugBank database (www.http://www.drugbank.ca/), containing 1442 drugs with measured log P between -9 and +10 (average value 2.0, standard deviation 2.2), only 2.1% of the molecules have log P values comprised between −4 and −3, while for 5.6% of the database this value ranges between −1 and −2.

Enzymatic inhibition assays performed on hNV and mNV-RdRps showed that **8** retains potent inhibition activity with IC_50_ values (*ca.* 30 nM for hNV-RdRp and *ca.* 60 nM for mNV) that are fully comparable with those of Suramin reported earlier [23]. Notably, the Suramin IC_50_ values previously reported were crosschecked in the present study, under the experimental conditions here reported, employing the same enzyme preparation used for assaying **6** and **8** inhibitions. The low nanomolar IC_50_ displayed by **8** is in keeping with its close structural relationship with Suramin. Moreover, the observation that **8** inhibitory potency *vs.* NV-RdRps was conserved (relative to Suramin) supports our structure-based hypothesis that the sulphonate group at position 3 of the naphthalene head (removed in **8**) is not involved in the RdRp recognition mechanism. Smaller Suramin-related compounds, isolated during the synthetic steps leading to **8**, were found to be less potent inhibitors, relative to Suramin (**9**) and **8** (Table 2). Nevertheless, the effects exerted to the targeted enzymes were sufficient to drive structural experiments. Since Suramin and **8** showed an almost identical IC_50_ value and close structural formulas, we focused on **6** whose smaller size may allow mapping new regions in the enzyme active site. In particular, **6** is a non-symmetric fragment of **8** hosting only one naphthalene disulphonate head. From a structural viewpoint, **6** can be seen as composed of two distinct parts: the naphthalene disulphonate acid head, which is involved in main interactions with the enzyme at the newly mapped binding site, and the tail region (composed of a toluene and a nitrobenzene ring linked by an amide group), which may provide additional interactions outside the main contact region. Inspection of the crystal structures showed that **6** binds to a site located in the polymerase thumb domain, more specifically in a cleft along the exit path for the newly synthesized RNA. This site doesn't match the previously identified Suramin binding site, but corresponds to the Pyridoxal-5′-phosphate-6-(2′-naphthylazo-6′-nitro-4′,8′-disulphonate; PPNDS) [32], [33] binding site [34](Tarantino *et al.* in press). Notably, PPNDS is also a molecule composed of two parts: the naphthalene disulphonate head and the pyridoxal phosphate group, linked by an azo bridge.

Our analysis on the NV-RdRp/**6** complex crystal structure shows that the sulphonate groups on the naphthalene sulphonic head interact with the RNA binding loop (residues 433–440). The crystal structures of hNV-RdRp/RNA complexes (3BSO and 3H5X) [35], [36] revealed significantly different locations in the RNA-binding loop relative to the free RdRp, showing specific RNA-induced conformational changes of this loop [37]. Binding of compound **6** might freeze the loop in hNV RdRp in the conformation found in the free enzyme hindering efficient RNA binding.

In the hNV-RdRp/**6** complex, the inhibitor electron density fades rapidly after the amido group linked to the naphthalene disulphonate moiety. The latter, in fact, is shifted toward His433 and tilted by steric hindrance with the Gln439/Gln414 “floor” (Fig. 3A,C), thus leaving the unmodeled part of **6** in an open enzyme region that fails to stabilize the ligand in one conformation. Thus, the lower inhibitory potency of **6** *vs.* hNV-RdRp may be linked to failure of establishing enzyme/inhibitor stabilizing interactions along the whole inhibitory molecule, which appears to remain mobile for about 50% of its scaffold.

On the contrary in the mNV-RdRp/**6** structure, the inhibitor could be completely modeled thanks to the different disposition of the naphthalene head due to the loss of the interaction with His433. The new interactions of **6** with Arg392 and Arg393 are in agreement with the higher potency of **6** *vs.* mNV-RdRp relative to hNV-RdRp. In contrast, **8** shows a slightly lower inhibitory effect on mNV relative to hNV-RdRp as observed for Suramin, probably because it binds to the same binding site of the latter inhibitor, achieving a different interaction network with the protein. The current crystal structure data, available only for the mNV-RdRp/Suramin complex (Mastrangelo *et al.*,2012) do not allow further comparisons.

The newly discovered NV-RdRP inhibitor binding site, mapped by **6** and by PPNDS, together with the different conformational behaviors displayed by **6** bound to hNV and mNV-RdRps, appear as two novel structural grounds to be exploited for the design and development of more potent/selective hNV-RdRp inhibitors.

## Conclusions

This study presented the design and synthesis of a modified Suramin molecule (**8**), the crystal structure analyses of NV-RdRps in complexes with a Suramin fragment (**6**), and the evaluation of inhibitory parameters for five related compounds targeting mNV and hNV-RdRps. A total synthesis of **8** from commercially available reagents was accomplished, which involved five steps with a 45% overall yield. In comparison with Suramin (**9**) bearing six hydrophilic sulphonate groups, the new and symmetric **8** contains four sulphonate groups, and exhibits the same potency as Suramin against NV-RdRps. However, its water solubility and log *P* value are more favorable compared to Suramin. In contrast, the tested reaction intermediates proved less effective in inhibiting NV polymerases.

Analysis of the crystal structures of hNV-RdRp/**6** complexes provided a mechanistic explanation of the decreased potency displayed by this compound. In fact, although the inhibitor naphthalene disulphonate ‘head’ is strongly bound to a specific pocket in the hNV-RdRp thumb domain, the rest of the molecule is hardly involved in enzyme/inhibitor interactions, negatively affecting **6** affinity for the polymerase active region. On the contrary, the higher inhibitory potency of **6** against mNV-RdRp correlates with a fully defined binding mode and interactions of the inhibitor to the RdRp active site. It may be noted that the related PPNDS NV-RdRp inhibitor, which displays a lower molecular weight and an overall more rigid structure, while adopting the same binding mode for the naphthalene disulphonate moiety, is involved in strong interactions with the enzyme in the naphthalene disulphonate substituent region, and displays low nM inhibitory potency [34] (Tarantino *et al*., in press). The basic structural and functional knowledge here reported for Suramin fragments, and particularly for **6**, will guide further Suramin chemical modifications aimed at the identification of hNV-RdRp inhibitors potentially able to be developed as anti-Norovirus agents.

## Materials and Methods

### General Procedure

All reactions were carried out in oven-dried glassware (120 °C) under an atmosphere of nitrogen unless otherwise indicated. Acetone, diethylether, toluene, acetic acid, and methanol were purchased from Mallinckrodt Chemical Co. 4-Amino-1,5-naphthalenesulphonate acid monosodium salt and triphosgene were purchased from TCI Chemical Co. 4-Methyl-3-nitrobenzoyl chloride was purchased from Sigma–Aldrich Chemical Co. Pd/C (10%) was purchased from Alfa Aesar Chemical Co. 3-Nitrobenzoyl chloride was purchased from Across Organics Chemical Co. Analytical thin layer chromatography (TLC) was performed on precoated plates (silica gel 60 F–254), purchased from Merck Inc. High performance liquid chromatography (HPLC) was performed on two Waters 515 HPLC Pumps equipped with a Waters 2489 UV/Visible Detector and a Thermo 5 μm Hypersil ODS (250×4.6 mm D.I.). Purity of products **3**, **4**, **6**, **7**, and **8** was >98.0%, as checked by HPLC. Infrared (IR) spectra were measured on a Perkin–Elmer model spectrum one B spectrophotometer and Perkin–Elmer Spectrum 100 FT-IR Spectrometer. Absorption intensities are recorded by the following abbreviations: s, strong; m, medium; w, weak. Proton NMR spectra were obtained on a Varian Mercury-400 (400 MHz) spectrometer or a Bruker AV-400 (400 MHz) by use of DMSO-*d*_6_ as solvent. Proton NMR chemical shifts are referenced to the DMSO-*d*_6_ quintet (*δ* 2.49 ppm). Carbon-13 NMR spectra were obtained on a Varian Mercury-400 (100 MHz) spectrometer or a Bruker AV-400 (100 MHz) by use of DMSO-*d*_6_ as solvent. Carbon-13 chemical shifts are referenced to the center of the DMSO-*d*_6_ septet (*δ* 39.5 ppm). Multiplicities are recorded by the following abbreviations: s, singlet; d, doublet; t, triplet; q, quartet; m, multiplet; *J*, coupling constant (hertz). High-resolution mass spectra were obtained by means of a VARIAN-901 mass spectrometer.

### 4-(4-Methyl-3-nitrobenzamido)naphthalene-1,5-disulphonate Acid Disodium Salt (3)

4-Amino-1,5-naphthalenedisulphonate acid monosodium salt (**2**, 0.998 g, 3.07 mmol, 1.0 equiv) was dissolved in water (12 ml) and the pH was adjusted to 4.0 by addition of aqueous Na_2_CO_3_ solution (2.0 M). To this solution 4-methyl-3-nitrobenzoyl chloride (**1**, 0.863 g, 4.33 mmol, 1.4 equiv) in toluene (2.7 ml) was slowly added. Its pH value was maintained continuously at 4.0 by addition of aqueous Na_2_CO_3_ solution (2.0 M). After the reaction mixture was stirred at room temperature for 12 hours, the toluene layer was separated out and the pH value of the aqueous layer was adjusted to 2.0. The aqueous layer was washed with diethyl ether (4×10 ml) and then pH was adjusted to 7.0 by addition of aqueous Na_2_CO_3_ solution (2.0 M). The water was removed under vacuum at 45 °C to give a crude solid, which was then dissolved in boiling methanol followed by hot filtration. The residue was dried over P_2_O_5_ under vacuum to give **3** (1.27 g, 2.49 mmol) in 81% yield, as a pale yellow powder: ^1^H NMR (DMSO-*d*_6_, 400 MHz) *δ* 12.81 (s, 1 H, NH), 9.10 (d, *J* = 8.8 Hz, 1 H, ArH), 8.66 (s, 1 H, ArH), 8.38 (d, *J* = 8.0 Hz, 1 H, ArH), 8.29 (d, *J* = 8.0 Hz, 1 H, ArH), 8.03-8.02 (m, 2 H, ArH), 7.66 (d, *J* = 8.0 Hz, 1 H, ArH), 7.47 (t, *J* = 8.0 Hz, 1 H, ArH), 2.60 (s, 3 H, CH_3_); ^13^C NMR (DMSO-*d*_6_, 100 MHz) *δ* 163.32 (C = O), 148.71, 141.65, 141.56, 135.61, 134.72, 133.70, 132.76, 132.32, 131.56, 130.83, 127.20, 124.46, 123.99, 123.54, 123.44, 122.51, 19.52; IR (KBr) 3359 (br, NH), 2918 (s), 1647 (m, C = O), 1547 (m), 1522 (m), 1346 (m), 1195 (s), 1044 (m) cm^−1^; MS (ESI) *m/z* (M+Na)^+^ 533, (M+H)^+^ 511; HRMS (ESI) calc. for (C_18_H_12_N_2_Na_2_O_9_S_2_+H)^+^: 510.9858, found 510.9845.

### 4-(3-Amino-4-methylbenzamido)naphthalene-1,5-disulphonate Acid Disodium Salt (4)

Nitro compound **3** (0.404 g, 0.792 mmol, 1.0 equiv) was dissolved in water (5.0 ml), to which 10% Pd/C (20.0 mg, 5.0% weight of **3**) was added. After the reaction mixture was stirred at room temperature for 12 hours under H_2_ atmosphere (4.0 bar), the Pd/C was filtered off by use of celite pad. Water was removed under vacuum at 45 °C to give a crude solid, which was then dissolved in boiling methanol followed by hot filtration. The residue was dried over P_2_O_5_ under vacuum to give **4** (0.347 g, 0.722 mmol) in 91% yield, as a brown powder: ^1^H NMR (DMSO-*d*_6_, 400 MHz) *δ* 12.37 (s, 1 H, NH), 9.08 (d, *J* = 8.4 Hz, 1 H, ArH), 8.29 (d, *J* = 7.2 Hz, 1 H, ArH), 8.03 (d, *J* = 8.4 Hz, 1 H, ArH), 7.97 (d, *J* = 8.4 Hz, 1 H, ArH), 7.46 (t, *J* = 8.4 Hz, 1 H, ArH), 7.31–7.28 (m, 2 H, ArH), 7.01 (d, *J* = 7.6 Hz, 1 H, ArH), 5.06 (br, 2 H, NH_2_), 2.12 (s, 3 H, CH_3_); ^13^C NMR (DMSO-*d*_6_, 100 MHz) *δ* 166.25 (C = O), 146.14, 141.89, 140.74, 134.85, 134.14, 131.56, 130.61, 129.32, 126.85, 124.48, 124.33, 123.42, 123.31, 122.10, 115.94, 113.93, 17.44; IR (KBr) 3445 (br, NH), 2918 (w), 1652 (m, C = O), 1575 (s), 1546 (s), 1525 (s), 1414 (m), 1344 (m), 1193 (s), 1073 (w), 1044 (s), 847 (w) cm^−1^; MS (ESI) *m/z* (M+Na)^+^ 503, (M+H)^+^ 481; HRMS (ESI) calcd for (C_18_H_14_N_2_Na_2_O_7_S_2_+H)^+^: 481.0116, found 481.0115.

### 4-[4-Methyl-3-(3-nitrobenzamido)benzamido]naphthalene-1,5-disulsulphonate Acid Disodium Salt (6)

Aniline derivative **4** (0.197 g, 0.410 mmol, 1.0 equiv) was dissolved in water (3.0 ml) and the pH was adjusted to 3.0 by addition of aqueous CH_3_CO_2_H solution. To this solution *m*-nitrobenzoyl chloride (**5**, 0.110 g, 0.593 mmol, 1.43 equiv) in toluene (0.60 ml) was slowly added. Its pH value was maintained again at 3.0 by addition of aqueous Na_2_CO_3_ solution (2.0 M). After the reaction mixture was stirred at room temperature for 12 hours, the toluene layer was separated out and the pH value of the aqueous layer was adjusted to 2.0. The aqueous layer was washed with diethyl ether (4×5 ml) and then pH was adjusted to 7.0 by addition of aqueous Na_2_CO_3_ solution (2.0 M). Water was removed under vacuum at 45 °C to give a crude solid, which was then dissolved in boiling methanol followed by hot filtration. The residue was dried over P_2_O_5_ under vacuum to give **6** (0.212 g, 0.337 mmol) in 82% yield as a yellow powder: ^1^H NMR (DMSO-*d*_6_, 400 MHz) *δ* 12.62 (s, 1 H, NH), 10.59 (s, 1 H, NH), 9.09 (d, *J* = 8.4 Hz, 1 H, ArH), 8.85 (s, 1 H, ArH), 8.50 (d, *J* = 8.0 Hz, 1 H, ArH), 8.45 (d, *J* = 8.4 Hz, 1 H, ArH), 8.29 (d, *J* = 7.2 Hz, 1 H, ArH), 8.05–7.99 (m, 4 H, ArH), 7.85 (t, *J* = 8.0 Hz, 1 H, ArH), 7.46 (t, *J* = 8.4 Hz, 1 H, ArH), 7.41 (d, *J* = 8.0 Hz, 1 H, ArH), 2.32 (s, 3 H, CH_3_); ^13^C NMR (DMSO-*d*_6_, 100 MHz) *δ* 64.93 (C = O), 163.30 (C = O), 147.82, 141.74, 141.11, 137.40, 137.38, 135.77, 134.36, 134.17, 133.71, 131.56, 130.71, 130.24, 130.04, 127.01, 126.78, 126.17, 125.87, 124.49, 123.46, 123.42, 122.55, 122.36, 18.03; IR (KBr) 3467 (br, NH), 1657 (m, C = O), 1580 (s), 1531 (m), 1417 (m), 1227 (m), 1190 (m), 1040 (m), 835 (w) cm^−1^; MS (ESI) *m/z* (M+Na)^+^ 652, (M+H)^+^ 630; HRMS (ESI) calcd for (C_25_H_17_N_3_Na_2_O_10_S_2_+H)^+^: 630.0229, found 630.0223.

### 4-[3-(3-Aminobenzamido)-4-methylbenzamido]naphthalene-1,5-disulphonate Acid Disodium Salt (7)

Nitro compound **6** (0.103 g, 0.164 mmol, 1.0 equiv) was dissolved in water (2.0 ml), to which 10% Pd/C (5.0 mg, 5.0% weight of **6**) was added. After the reaction mixture was stirred at room temperature for 12 hours under H_2_ atmosphere (4.0 bar), the Pd/C was filtered off by use of celite pad. Water was removed under vacuum at 45 °C to give a crude solid, which was then dissolved in boiling methanol followed by hot filtration. The residue was dried over P_2_O_5_ under vacuum to give **7** (89.1 mg, 0.148 mmol) in 90% yield, as a brown powder: ^1^H NMR (DMSO-*d*_6_, 400 MHz) *δ* 12.60 (s, 1 H, NH), 9.85 (s, 1 H, NH), 9.09 (d, *J* = 8.4 Hz, 1 H, ArH), 8.29 (d, *J* = 7.2 Hz, 1 H, ArH), 8.04–7.99 (m, 3 H, ArH), 7.96 (s, 1 H, ArH), 7.45 (t, *J* = 8.0 Hz, 1 H, ArH), 7.36 (d, *J* = 8.0 Hz, 1 H, ArH), 7.17 (s, 1 H, ArH), 7.14–7.13 (m, 2 H, ArH), 6.76–6.73 (m, 1 H, ArH), 5.30 (br, 2 H, NH_2_), 2.31 (s, 3 H, CH_3_); ^13^C NMR (DMSO-*d*_6_, 100 MHz) *δ* 166.18 (C = O), 165.13 (C = O), 148.81, 141.76, 141.03, 137.19, 136.52, 135.38, 134.48, 133.56, 131.58, 130.72, 129.86, 128.78, 127.04, 126.65, 125.30, 125.27, 124.55, 123.48, 122.38, 116.81, 114.78, 113.18, 18.03; IR (KBr) 3356 (br, NH), 3001 (w), 1575 (s), 1423 (m), 1335 (w), 1316 (w), 1223 (m), 1204 (m), 1190 (m), 1042 (m), 836 (w) cm^−1^; MS (ESI) *m/z* (M+Na)^+^ 622, (M+H)^+^ 600; HRMS (ESI) calcd for (C_25_H_19_N_3_Na_2_O_8_S_2_+H)^+^: 600.0487, found 600.0487.

### 4-4′-(Carbonylbis[imino-3,1-phenylenecarbonylimino(4-methyl-3,1-phenylene) carbonylimino])bis-1,5-naphthalenedisulphonate Acid Tetrasodium Salt (8)

Aniline derivative **7** (49.4 mg, 82.4 μmol, 2.0 equiv) was dissolved in water (2.0 ml) and the pH was adjusted to 3.0 by addition of aqueous Na_2_CO_3_ solution (2.0 M). To this solution triphosgene (33.0 mg, 0.111 mmol, 1.3 equiv) in toluene (0.50 ml) was slowly added. Its pH value was maintained again at 3.0 by addition of aqueous Na_2_CO_3_ solution (2.0 M). After the reaction mixture was stirred at room temperature for 12 hours, the toluene layer was discarded. Water was removed under vacuum at 45 °C to give a crude solid, which was then dissolved in boiling methanol followed by hot filtration. The residue was dried over P_2_O_5_ under vacuum to give **8** (42.4 mg, 34.0 μmol) in 83% yield as a white powder: ^1^H NMR (DMSO-*d*_6_, 400 MHz) *δ* 12.60 (s, 2 H, NH), 11.08 (s, 2 H, NH), 10.07 (s, 2 H, NH), 9.09 (d, *J* = 8.4 Hz, 2 H, ArH), 8.29 (d, *J* = 7.2 Hz, 2 H, ArH), 8.10 (s, 2 H, ArH), 8.057.98 (m, 8 H, ArH), 7.82 (d, 2 H, *J* = 8.8 Hz, ArH), 7.57 (d, *J* = 8.0 Hz, 2 H, ArH), 7.45 (t, *J* = 8.0 Hz, 2 H, ArH), 7.41–7.37 (m, 4 H, ArH), 2.32 (s, 6 H, 2×CH_3_); ^13^C NMR (DMSO-*d*_6_, 100 MHz) *δ* 165.81 (C = O), 165.06 (C = O), 153.53 (C = O), 141.76, 141.10, 137.18, 136.43, 135.17, 134.42, 133.60, 131.57, 130.72, 129.89, 128.48, 127.01, 126.60, 125.32, 124.51, 123.47, 123.41, 122.36, 121.10, 120.19, 117.60, 114.19, 18.06; IR (KBr) 3355 (br, NH), 2919 (s), 2848 (m), 1652 (s, C = O), 1634 (s, C = O), 1574 (m), 1422 (m), 1318 (m), 1224 (w), 1191 (w), 1086 (w), 1039 (w), 828 (w) cm^−1^; MS (ESI) *m/z* (M+2 Na)^2+^ 635; HRMS (ESI) calcd for (C_51_H_36_N_6_Na_4_O_17_S_4_+2 Na)^2+^: 635.0197, found 635.0116.

### Water Solubility

A stock solution was prepared by dissolving a precisely weighed amount of Suramin derivatives in deionized water (1.0 ml). The UV absorption maximum of each compound was measured by dilution of the solution with deionized water as necessary. A saturated solution of each compound was then prepared by use of deionized water (1.0 ml) in the presence of an excess of Suramin derivatives for 1.0 hour. The obtained saturated solution was filtered to remove solid compound through a Millipore filter (0.45 μm) and was scanned by UV spectroscopy at the wavelength of the absorption maximum previously determined.

### Partition Coefficient

The *n*-octanol/water partition coefficient of Suramin derivatives was determined by the shake-flask method. *n*-Octanol and pH 7.4 phosphate buffer were mutually saturated for 4.0 hours, and the phases were separated. A stock solution of each Suramin derivative was prepared using pH 7.4 phosphate buffer solution. Suramin derivatives were partitioned between *n*-octanol and pH 7.4 phosphate buffer. Then the phase mixtures were shaken for 1 hour at constant 25 °C. After separation, the absorbance of the phosphate buffer solutions (pH 7.4) was measured by UV spectrophotometry. Three replicates were performed for each compound. The *P* value corresponded to the quotient between *n*-octanol and buffer concentrations of the drug. The log *P* values were an average of three independent experiments.

### Expression and purification of the hNV and mNV-RdRps

The NV-RdRps were expressed and purified as previously described [23], and stocked in 25 mM Tris/HCl pH 7.4, 1 mM DTT, 100 mM NaCl, 1 mM EDTA. Data and/or materials of NV-RdRps are available for sharing or collaboration following signing of a material transfer agreement with Riboxx GmbH (Germany).

### *In-vitro* RdRp inhibition assays

RdRp assays were performed as previously described [23]. In brief poly(C) (MP Biomedicals) was used as template annealed with oligoG12 as primer (62.5 nM final concentration) and GTP (100 μM final concentration) as substrate, in a 200 μl reaction mixture containing 20 mM Tris/HCl pH 7.5, 25 mM NaCl, 5 mM MgCl_2_, 0.3 mM MnCl_2_, 1 mM DTT, PicoGreen Quantitation Reagent (Molecular Probes) diluted 1/200 20 U ml−1 RiboLock Ribonuclease Inhibitor (Fermentas). Reactions were started by the addition of GTP following the fluorescence of samples in a fluorescence reader (Varian, Cary Eclipse Fluorescence Spectrophotometer).

### Thermofluorometric characterization of the hNV-RdRp inhibitors interaction

Thermofluorimetric (Thermal shift) assays for the evaluation of the hNV-RdRp melting temperature (T_m_) in the absence/presence of the inhibitors, were conducted in a MiniOpticon Real Time PCR Detection System (Bio-Rad), using the fluorescent dye Sypro Orange. Solutions of 4 μl of the NV-RdRp domain (final hNV-RdRp concentration 7 μM, final mNV-RdRp concentration 1.6 μM) were diluted in 9.5 μl of its buffer, and mixed with 3.5 μl of Sypro orange (Sigma) diluted 60×, and 1 μl of **6** or **8** (4 μM final concentration). In control samples the inhibitors were replaced by water. The sample plates were heated from 20 to 90°C with a heating rate of 0.2 °C/min. Fluorescence intensities were measured within excitation/emission ranges of 470–505 nm and 540–700 nm, respectively.

### Crystallization of the NV-RdRps in presence of 6

Sitting drop crystallization experiments on hNV-RdRp (11 mg/ml) were prepared using an Oryx-8 crystallization robot (Douglas Instruments, East Garston, UK), from a 50% mixture of the protein with the reservoir solution (final drop volume 0.3 μl). Crystals were obtained after 4 weeks at 20°C, in 1.2 M Na citrate, 100 mM Na cacodylate pH 6.2, and NaCl 125 mM. Before X-ray data collection, crystals were soaked in a cryoprotectant solution (1.4 M Na citrate, 100 mM Na cacodylate pH 6.2, and 25% glycerol) with 10 mM **6**, in the presence of 62.5 nM dsRNA and 100 μM GTP for 36 hours, then flash-cooled in liquid nitrogen. The hNV-RdRp/**6** crystals diffracted to a maximum resolution of 2.02 Å at the ESRF Synchrotron (Grenoble, France) beam line ID29. X-ray diffraction data were indexed using MOSFLM [38], and intensities were merged using SCALA [39]. Microbatch crystallization experiments on mNV-RdRp were prepared using an Oryx-8 crystallization robot (Douglas Instruments, East Garston, UK), from a 2∶1∶1 mixture (drop volume 0.4 μl) of protein (10 mg/ml), precipitant, and 100 mM MgCl_2_ (25 mM in the drop), covered by Al's oil (a mixture of 50% Paraffin oil and 50% Silicon oil). Prismatic crystals of approximately 150×80×30 μm^3^ were obtained after 1 week at 20°C, in 1.6 M (NH_4_)_2_SO_4_, 12% glycerol, 100 mM TRIS-HCl pH 8.4. Before X-ray data collection, crystals were soaked in a cryoprotectant solution (1.8 M (NH_4_)_2_SO_4_, 100 mM TRIS-HCl pH 8.4, and 25% glycerol) with 20 mM of **6**, in the presence of 62.5 nM of dsRNA and 100 μM of GTP for 36 hours, than flash-cooled in liquid nitrogen. dsRNA and GTP were added to the soaking solution as additives to increase chances to obtain ligand-protein complexes The mNV-RdRp/**6** crystals diffracted to a maximum resolution of 2.3 Å at the ESRF Synchrotron (Grenoble, France) beam line ID29. X-ray diffraction data were indexed using MOSFLM, and intensities were merged using SCALA [39].

### Structure determination and refinement

The three-dimensional structures of hNV-RdRp and mNV-RdRp in the complexes with **6** were solved by the Molecular Replacement method using the program MOLREP [40] and as search models the 3D structures of the respective ligand-free RdRps (PDB-id 2B43 and id 3UQS, respectively). The crystal asymmetric unit molecule(s) were individually subjected to rigid-body refinement, and subsequently to restrained refinement using REFMAC5 [41]. A random set comprising 5% of the data was omitted from refinement for R-free calculation. Manual rebuilding with COOT [42] and additional refinement with BUSTER [43] and REFMAC5 were subsequently performed, as needed. Data collection, refinement statistics as well as stereochemical quality of the models are summarized in Table 3. Atomic coordinates and structure Factor files for hNV-RdRp/**6** and mNV-RdRp/**6** complexes have been deposited with the Protein Data Banks as entries 4NRT and 4NRU, respectively.

## References

[1] Clarke IN, Lambden PR (1997) The molecular biology of caliciviruses. J Gen Virol 78 (Pt 2): 291–301. 9018049 10.1099/0022-1317-78-2-291

[2] Patel MM, Widdowson M-A, Glass RI, Akazawa K, Vinjé J, et al (2008) Systematic literature review of role of noroviruses in sporadic gastroenteritis. Emerg Infect Dis 14: 1224–1231. 18680645 10.3201/eid1408.071114 PMC2600393

[3] Atmar RL, Estes MK (2012) Norovirus vaccine development: next steps. Expert Rev vaccines 11: 1023–1025. 23151158 10.1586/erv.12.78 PMC3757541

[4] Bartsch SM, Lopman BA, Hall AJ, Parashar UD, Lee BY (2012) The potential economic value of a human norovirus vaccine for the United States. Vaccine 30: 7097–7104. 23026689 10.1016/j.vaccine.2012.09.040 PMC3517973

[5] Cheetham S, Souza M, Meulia T, Grimes S, Han MG, et al (2006) Pathogenesis of a genogroup II human norovirus in gnotobiotic pigs. J Virol 80: 10372–10381. 17041218 10.1128/JVI.00809-06 PMC1641747

[6] Souza M, Cheetham SM, Azevedo MSP, Costantini V, Saif LJ (2007) Cytokine and antibody responses in gnotobiotic pigs after infection with human norovirus genogroup II.4 (HS66 strain). J Virol 81: 9183–9192. 17581999 10.1128/JVI.00558-07 PMC1951422

[7] Bok K, Parra GI, Mitra T, Abente E, Shaver CK, et al (2011) Chimpanzees as an animal model for human norovirus infection and vaccine development. Proc Natl Acad Sci United States Am 108: 325–330. 10.1073/pnas.1014577107 PMC3017165 21173246

[8] Karst SM (2010) Pathogenesis of noroviruses, emerging RNA viruses. Viruses 2: 748–781. 21994656 10.3390/v2030748 PMC3185648

[9] Clarke IN, Lambden PR (2000) Organization and expression of calicivirus genes. J Infect Dis 181. 10.1086/315575 10804143

[10] Hansman GS, Matsubara N, Oka T, Ogawa S, Natori K, et al (2005) Deletion analysis of the sapovirus VP1 gene for the assembly of virus-like particles. Arch Virol 150: 2529–2538. 16052282 10.1007/s00705-005-0599-5

[11] Hansman GS, Jiang XJ, Green KY (2010) Caliciviruses: Molecular and Cellular Virology. Caister Academic Press.

[12] Fullerton SWB, Blaschke M, Coutard B, Gebhardt J, Gorbalenya A, et al (2007) Structural and functional characterization of sapovirus RNA-dependent RNA polymerase. J Virol 81: 1858–1871. 17121797 10.1128/JVI.01462-06 PMC1797576

[13] Hawking F (1978) Suramin: with special reference to onchocerciasis. Adv Pharmacol Chemother 15: 289–322. 358805 10.1016/s1054-3589(08)60486-x

[14] Mitsuya H, Matsushita S, Yarchoan R, Broder S (1984) Protection of T cells against infectivity and cytopathic effect of HTLV-III in vitro. Princess Takamatsu Symp 15: 277–288. 6085846

[15] Dunn PM, Blakeley AG (1988) Suramin: a reversible P2-purinoceptor antagonist in the mouse vas deferens. Br J Pharmacol 93: 243–245. 3359103 10.1111/j.1476-5381.1988.tb11427.x PMC1853806

[16] Kassack MU, Braun K, Ganso M, Ullmann H, Nickel P, et al (2004) Structure-activity relationships of analogues of NF449 confirm NF449 as the most potent and selective known P2X1 receptor antagonist. Eur J Med Chem 39: 345–357. 15072843 10.1016/j.ejmech.2004.01.007

[17] Zamai M, Hariharan C, Pines D, Safran M, Yayon A, et al (2002) Nature of Interaction between basic fibroblast growth factor and the antiangiogenic drug 7,7-(carbonyl-bis[imino-N-methyl-4,2-pyrrolecarbonylimino[N-methyl-4,2-pyrrole]-carbonylimino])-bis-(1,3-naphtalene disulfonate). II. Removal of polar interactions affects protein folding. Biophys J 82: 2652–2664. 11964252 10.1016/S0006-3495(02)75607-5 PMC1302054

[18] Sola F, Farao M, Pesenti E, Marsiglio A, Mongelli N, et al (1995) Antitumor activity of FCE 26644 a new growth-factor complexing molecule. Cancer Chemother Pharmacol 36: 217–222. 7781141 10.1007/BF00685849

[19] Jagielski AK, KryÅ>kiewicz E, BryÅ‚a J (2006) Suramin-induced reciprocal changes in glucose and lactate synthesis in renal tubules contribute to its hyperglycaemic action. Eur J Pharmacol 537: 205–209. 16626687 10.1016/j.ejphar.2006.03.029

[20] Kaur M, Reed E, Sartor O, Dahut W, Figg WD (2002) Suramin's development: what did we learn? Investig new drugs 20: 209–219. 12099581 10.1023/a:1015666024386

[21] Lustberg MB, Pant S, Ruppert AS, Shen T, Wei Y, et al (2012) Phase I/II trial of non-cytotoxic suramin in combination with weekly paclitaxel in metastatic breast cancer treated with prior taxanes. Cancer Chemother Pharmacol 70: 49–56. 22729159 10.1007/s00280-012-1887-x PMC3466596

[22] Kaplan LD, Wolfe PR, Volberding PA, Feorino P, Levy JA, et al (1987) Lack of response to suramin in patients with AIDS and AIDS-related complex. Am J Med 82: 615–620. 3548350 10.1016/0002-9343(87)90108-2

[23] Mastrangelo E, Pezzullo M, Tarantino D, Petazzi R, Germani F, et al (2012) Structure-based inhibition of Norovirus RNA-dependent RNA polymerases. J Mol Biol 419: 198–210. 22446684 10.1016/j.jmb.2012.03.008

[24] Thomas G (n.d.) Medicinal chemistry. Chichester; Hoboken, NJ: John Wiley.

[25] Camp D, Davis RA, Campitelli M, Ebdon J, Quinn RJ (2012) Drug-like properties: guiding principles for the design of natural product libraries. J Nat Prod 75: 72–81. 22204643 10.1021/np200687v

[26] Padiya KJ, Gavade S, Kardile B, Tiwari M, Bajare S, et al (2012) Unprecedented “In Water” imidazole carbonylation: paradigm shift for preparation of urea and carbamate. Org Lett 14: 2814–2817. 22594942 10.1021/ol301009d

[27] Bookser BC, Ugarkar BG, Matelich MC, Lemus RH, Allan M, et al (2005) Adenosine kinase inhibitors. 6. Synthesis, water solubility, and antinociceptive activity of 5-phenyl-7-(5-deoxy-beta-D-ribofuranosyl)pyrrolo[2,3-d]pyrimidines substituted at C4 with glycinamides and related compounds. J Med Chem 48: 7808–7820. 16302820 10.1021/jm050394a

[28] Kraszni M, Bányai I, Noszál B (2003) Determination of conformer-specific partition coefficients in octanol/water systems. J Med Chem 46: 2241–2245. 12747795 10.1021/jm030767c

[29] Lee S, Cho K-H, Acree WE, No KT (2012) Development of surface-SFED models for polar solvents. J Chem Inf Model 52: 440–448. 22242933 10.1021/ci2004913

[30] Eltahla AA, Lackovic K, Marquis C, Eden J-S, White PA (2013) A fluorescence-based high-throughput screen to identify small compound inhibitors of the genotype 3a hepatitis C virus RNA polymerase. J Biomol Screen 18: 1027–1034. 23708123 10.1177/1087057113489883

[31] Pinnen F, Cacciatore I, Cornacchia C, Sozio P, Iannitelli A, et al (2007) Synthesis and study of L-dopa-glutathione codrugs as new anti-Parkinson agents with free radical scavenging properties. J Med Chem 50: 2506–2515. 17451233 10.1021/jm070037v

[32] Wood CR, Hennessey TM (2003) PPNDS is an agonist, not an antagonist, for the ATP receptor of Paramecium. J Exp Biol 206: 627–636. 12502783 10.1242/jeb.00105

[33] Suzuki E, Kessler M, Montgomery K, Arai AC (2004) Divergent effects of the purinoceptor antagonists suramin and pyridoxal-5′-phosphate-6-(2′-naphthylazo-6′-nitro-4′,8′-disulfonate) (PPNDS) on alpha-amino-3-hydroxy-5-methyl-4-isoxazolepropionic acid (AMPA) receptors. Mol Pharmacol 66: 1738–1747. 15448189 10.1124/mol.104.003038

[34] Tarantino D, Pezzullo M, Mastrangelo E, Croci R, Rohayem J, et al.. (2013) Naphtalene-sulfonate inhibitors of human norovirus RNA-dependent RNA-polymerase. Antivir Res. 10.1016/j.antiviral.2013.11.016 24316032

[35] Zamyatkin DF, Parra F, Alonso JMM, Harki DA, Peterson BR, et al (2008) Structural insights into mechanisms of catalysis and inhibition in Norwalk virus polymerase. J Biol Chem 283: 7705–7712. 18184655 10.1074/jbc.M709563200

[36] Zamyatkin DF, Parra F, MachÃ­n A, Grochulski P, Ng KK-S (2009) Binding of 2′-amino-2′-deoxycytidine-5′-triphosphate to norovirus polymerase induces rearrangement of the active site. J Mol Biol 390: 10–16. 19426741 10.1016/j.jmb.2009.04.069

[37] Lee J-H, Alam I, Han KR, Cho S, Shin S, et al (2011) Crystal structures of murine norovirus-1 RNA-dependent RNA polymerase. J Gen Virol 92: 1607–1616. 21471315 10.1099/vir.0.031104-0

[38] Leslie AG, Powell HR (2007) Processing diffraction data with Mosflm. Evolving methods for macromolecular crystallography. Springer. pp. 41–51.

[39] Evans P (2006) Scaling and assessment of data quality. Acta Crystallogr D Biol Crystallogr 62: 72–82. 16369096 10.1107/S0907444905036693

[40] Vagin A, Teplyakov A (2010) Molecular replacement with MOLREP. Acta Crystallogr D Biol Crystallogr 66: 22–25. 20057045 10.1107/S0907444909042589

[41] Steiner RA, Lebedev AA, Murshudov GN (2003) Fisher's information in maximum-likelihood macromolecular crystallographic refinement. Acta Crystallogr D Biol Crystallogr 59: 2114–2124. 14646069 10.1107/s0907444903018675

[42] Emsley P, Lohkamp B, Scott WG, Cowtan K (2010) Features and development of Coot. Acta Crystallogr D Biol Crystallogr 66: 486–501. 20383002 10.1107/S0907444910007493 PMC2852313

[43] Smart OS, Womack TO, Flensburg C, Keller P, Paciorek W, et al (2012) Exploiting structure similarity in refinement: automated NCS and target-structure restraints in BUSTER. Acta Crystallogr D Biol Crystallogr 68: 368–380. 22505257 10.1107/S0907444911056058 PMC3322596

